# Clinical impact of Fn-induced high expression of KIR2DL1 in CD8 T lymphocytes in oesophageal squamous cell carcinoma

**DOI:** 10.1080/07853890.2021.2016942

**Published:** 2021-12-22

**Authors:** Xiaopeng Wang, Yiwen Liu, Yannan Lu, Simo Chen, Yaoping Xing, Haijun Yang, Xiaojun Wang, Yaowen Zhang, Tao Pan, Junkuo Li, Min Wang, Ning Zhang, Mengxia Liang, Fuyou Zhou

**Affiliations:** a Anyang Tumor Hospital (The Fourth Affiliated Hospital of Henan University of Science and Technology), Anyang, Henan, China; b The Third Affiliated Hospital of Xinxiang Medical University, Xinxiang, Henan, China; c College of Clinical Medicine, Laboratory of Molecular Biology of the First Affiliated Hospital, Cancer Institute of Henan University of Science and Technology, Henan Key Laboratory of Cancer Epigenetics, Luoyang, Henan, China; d Graduate School of Dalian Medical University, Dalian, Liaoning, China; e Basic Medical School of Henan University of Science and Technology, Luoyang, Henan, China; f Department of Lymphoma & Hematology, Hunan Cancer Hospital, The Affiliated Cancer Hospital of Xiangya School of Medicine, Central South University, Changsha, Hunan, China

**Keywords:** *Fusobacterium nucleatum*, oesophageal squamous cell carcinomas, CD8^+^ T lymphocytes, KIR2DL1, prognosis

## Abstract

**Background:**

To analyze the correlation between the inducing effect of *Fusobacterium nucleatum* (Fn) on the surface expression of the inhibitory receptor KIR2DL1 on CD8^+^ T cells in oesophageal squamous cell carcinoma (ESCC) and the clinicopathological features and survival prognosis and to explore its clinical significance.

**Methods:**

The inducing effect of Fn on CD8^+^ T cell surface inhibitory receptor KIR2DL1 expression was analyzed in a coculture system of human CD8^+^ T cells and ESCC cells infected with Fn. Fn infection and the expression of KIR2DL1 on CD8^+^ T cells were detected by RNAscope and immunohistochemistry in ESCC tissues, and the correlations between the inducing effect of Fn on KIR2DL1 expression on CD8^+^ T cells and clinicopathological features were analyzed. COX regression was used to analyze the influence of each factor on the prognosis of ESCC. Survival curves were plotted by the Kaplan–Meier method, and the effect of KIR2DL1 induction on survival time was analyzed by the log-rank test.

**Results:**

In the coculture system, KIR2DL1 expression on the surface of CD8^+^ T cells increased with increasing Fn infection time. In ESCC tissues, Fn infection was significantly correlated with high KIR2DL1 expression on CD8^+^ T cells. The Fn + CD8^+^KIR2DL1 positive patients were predominantly males who were smokers and alcohol drinkers. Moreover, patients with Fn infection were characterized by poor tumour differentiation, advanced clinical stage, and a short survival time. Meanwhile, Fn + CD8^+^KIR2DL1 positive group was independent risk factor affecting the prognosis of ESCC patients.

**Conclusions:**

Long-term drinking and smoking lead to an extremely unhealthy oral environment in which Fn infection and colonization are more likely to occur, thus inducing high expression of KIR2DL1 on the surface of CD8^+^ T cells, which can weaken the antitumour immune response and promote the malignant progression of ESCC.

**HIGHLIGHTS**
- Fn induced high expression of KIR2DL1 CD8^+^ T cells in a time-dependent manner.
- Fn can reduce the response of tumour cells to CDDP.
- The inducing effect of Fn on CD8^+^ T cell surface KIR2DL1 expression was significantly associated with the poor prognosis of ESCC patients.

## 1. Introduction

Oesophageal cancer is one of the most common malignant tumours in China, and more than 90% of oesophageal cancers are oesophageal squamous cell carcinoma (ESCC) [1]. The cause of ESCC has not been fully identified. *Fusobacterium nucleatum* (Fn) is an opportunistic oral pathogen [2]. Recent studies have indicated that Fn is intimately related to the development of ESCC [3,4]. Long-term colonization with Fn can not only enhance the proliferation and invasion abilities of cancer cells but also remodel the tumour microenvironment and inhibit the immune response [5,6]. The well-known killer cell immunoglobulin receptor (KIR) family belongs to the immunoglobulin superfamily [7], and the KIR family member KIR2DL1 is an important inhibitory molecule on the surface of CD8^+^ T cells. KIR2DL1-mediated immunosuppression is one of the causes of tumour immune escape and a major obstacle to successful tumour immunotherapy [8]. The expression of KIR2DL1 on CD8^+^ T cells plays an important role in regulating the immune response [8] and is positively correlated with the occurrence and development of tumours. Clinical data show that a variety of pathogenic microorganisms can induce high expression of inhibitory receptors on CD8^+^ T cells [9–11], leading to tumour cell escape from immune surveillance. Therefore, it is speculated that Fn may induce CD8^+^ T cells to express high levels of KIR2DL1, preventing them from being recognized and cleared by immune cells and promoting the malignant progression of ESCC.

In this study, the inducing effect of Fn on KIR2DL1 expression on the surface of CD8^+^ T cells was initially analyzed through a coculture system of human CD8^+^ T lymphocytes and ESCC cells infected with Fn. Fn infection in ESCC tissues and KIR2DL1 expression on CD8^+^ T cells were detected by an RNAscope assay and immunohistochemistry (IHC), respectively, to further analyze the correlations between the inducing effect of Fn and patients' clinicopathological characteristics and survival outcomes. Our findings provide new ideas and suggest new methods for ESCC treatment.

## 2. Materials and methods

### 2.1. Patients

**Figure 1.**
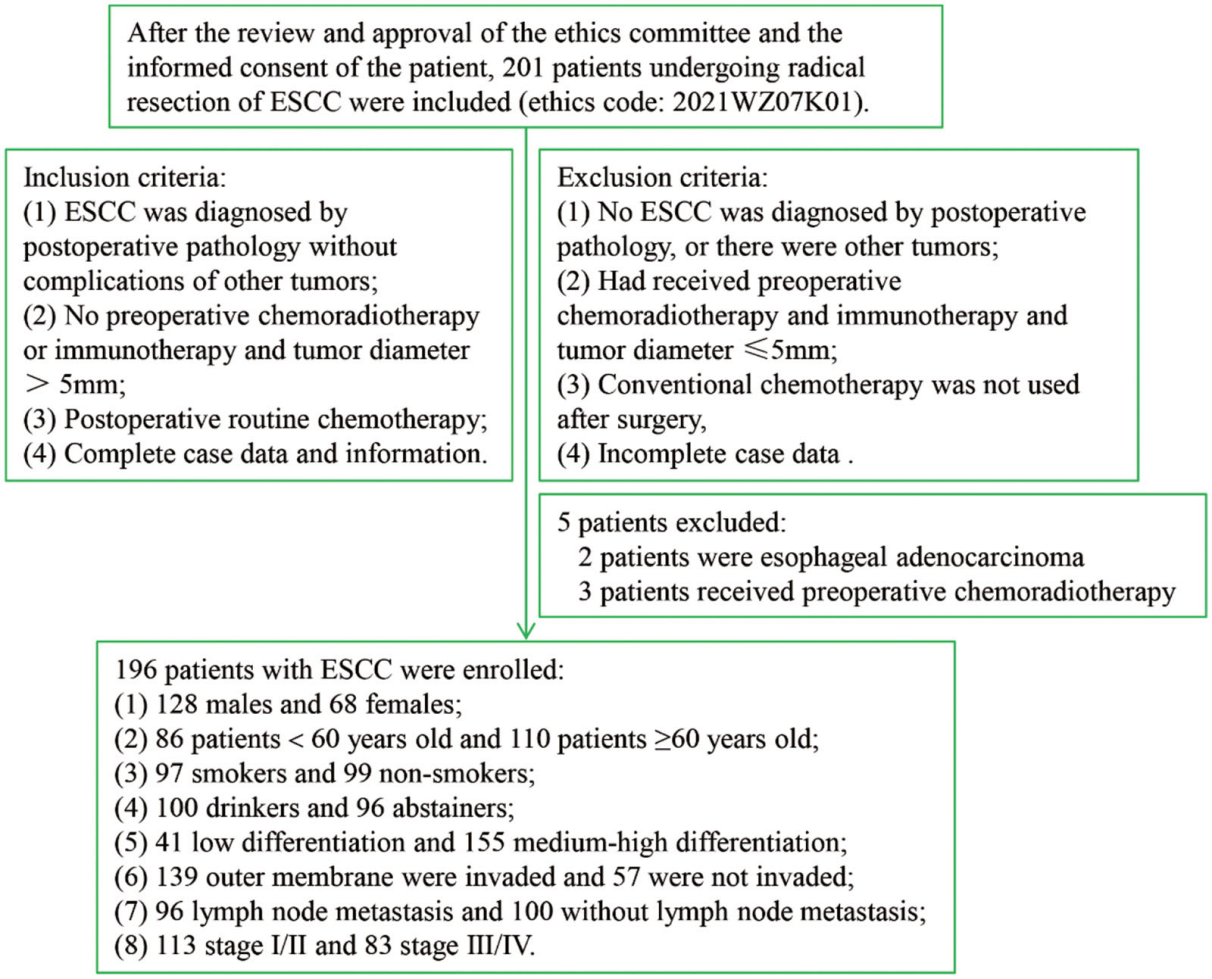
Enrolment criteria and clinicopathological data of 196 ESCC patients.

For this study, a total of 201 patients with ESCC treated at Anyang Tumour Hospital (ATH; Anyang, Henan, China) between January and December 2014 were enrolled after radical resection. The inclusion criteria are shown in Figure 1. A total of 196 patients with ESCC were ultimately included in the study. Patients who were still alive at 60 months of follow-up were excluded, and those who died due to ESCC were not excluded. This study was approved by Anyang Cancer Hospital and the hospital Ethics Committee, and the patients' written informed consent was obtained before surgery to participate in the study (ethics code: 2021WZ07K01).

### 2.2. Coculture of human CD8^+^ T cells with ESCC cells

The peripheral blood of healthy persons (volunteers in our laboratory) was put into anticoagulant tubes. After 1:1 dilution with normal saline, the blood was slowly spread on the surface of the appropriate Ficoll-Paque separation medium (GE). Centrifugation was performed at 800 × g for 20 min. The white film was carefully absorbed after stratification. Centrifugation at 800 g for 5 min. Precipitation of human peripheral blood mononuclear cells. CD8^+^ T lymphocytes were isolated from human peripheral blood monocytes with a magnetic bead sorting kit (Miltenyi). *In vitro* expansion was carried out in TexMACS medium (Miltenyi) containing hIL2 (50 U/mL, Miltenyi) and hIL-7-FC (70 ng/mL, Miltenyi) in a 5% CO_2_ incubator at 37 °C. For the specific steps, please refer to the previous publication of our research group [12]. KYSE150 cells were conventionally cultured in RPMI 1640 medium (containing 10% FBS, Gibco). When the confluence was 60–70%, CD8^+^ T lymphocytes were added to the cell culture medium at a ratio of 10:1 CD8^+^ T lymphocytes: KYSE150 cells and cocultured at 37 °C in a 5% CO_2_ incubator.

### 2.3. Fn infection of ESCC cells

Fn (Fn standard strain ATCC 25586, donated by the University of Louisville, USA) was cultured in a brain–heart infusion medium (Solarbio) containing 5% sterile defibrinated sheep blood (Solarbio), 1% haem chloride (Solarbio), and 0.1% vitamin K1 (Solarbio) at 37 °C in an anaerobic workstation (COY) containing 85% N_2_, 10% H_2_, and 5% CO_2_. The purity of Fn was detected by gram staining and culture on Columbia blood agar plates [12]. The Fn bacterial solution (OD600 = 1–2) with better viability was selected. When the cell confluence was 60–70%, Fn was added into the cell culture medium at an MOI of 10. Different times of infection (24 or 48 h) were used. A follow-up experiment was performed.

### 2.4. In vivo experiment

Twenty-four NSG mice were fed under specific pathogen-free (SPF) conditions. The source of the mice and specific feeding conditions were based on previous studies by our group [12]. The NSG mice were divided into four groups: ① control group; ② cisplatin (CDDP) treatment group; ③ Fn group; and ④ Fn + CDDP treatment group. KYSE150 cells were labelled with the fluorochrome PKH67. A total of 1 × 10^6^ KYSE150 cells were inoculated into the right axillae of the mice in groups ① and ②. A total of 1 × 10^6^ KYSE150 cells 24 h after Fn infection were inoculated into the right axillae of the mice in groups ③ and ④. The mice in groups ② and ④ were injected with CDDP every 3 days. After the tumours grew to 0.5 cm in diameter, 2 × 10^6^ human peripheral blood-derived CD8^+^ T lymphocytes were injected into the tail veins of all 24 NSG mice [12]. The tumours in the mice were photographed and measured with a small animal imager (PE). Four weeks later, the mice were sacrificed by cervical dislocation, and the tumour bodies were excised and weighed. The animal study was carried out in accordance with the Anyang Tumour Hospital animal care guidelines (ethics code: 2021WZ07K01).

### 2.5. RNAscope assay

Pathological sections (2 µm thick) of paraffin-embedded carcinoma tissue and adjacent tissues from each patient were prepared. After routine dewaxing and hydration, a target repair solution (ACD) was used for antigen retrieval. After incubation with hydrogen peroxide and protease Plus (ACD), Fn-specific probe hybridization (ACD) and RNAscope colour detection (ACD) were performed. Refer to instructions and literature for specific experimental steps [13]. The Fn (16S rRNA) signal was located in the cytoplasm of tumour cells and had a red granular appearance. A light microscope (Nikon) was used to count the number of red particles in 20 tumour cells under 400× magnification. The average number of particles counted in each of the 196 samples was used as the threshold for identifying positive cells: cells with several red particles greater than this threshold were considered positive cells. The positive rate in each specimen was calculated, and the average of the positive rates was used as the threshold for identifying the positive specimens. If the value was greater than this threshold, the specimen was considered positive. In this experiment, cells with at least eight single red particles of Fn or clusters of signal-positive cells were considered positive cells. A specimen with ≥30% positive cells in each section was considered a positive specimen, and the scoring details are shown in the references [13].

### 2.6. IHC

Pathological sections (2 µm thick) of paraffin-embedded carcinoma tissues and adjacent tissues from the same batch of patients were prepared. After conventional dewaxing and hydration, citrate buffer (Solarbio) was used for antigen retrieval. Peroxidase Blocker (ZSGB-BIO) and Goat Serum Blocker (ZSGB-BIO) were used. Two serial sections were obtained, anti-CD8 and anti-KIR2DL1 antibodies (50 μL of each) were added, and the sections were incubated at 4 °C overnight. After incubation with goat anti-rat or anti-rabbit (ZSGB-BIO) polymer and biotinylated anti-streptavidin antibodies in Peroxidase Blocker (ZSGB-BIO), DAB (Solarbio) chromogen was added. The sections were restained with haematoxylin (Solarbio), dehydrated with ethanol, cleared with xylene, and sealed with neutral gum. Refer to the manufacturer’s instructions and literature for the specific experimental steps [3]. The colocalization of CD8 and KIR2DL1 expression in two serial sections were observed by randomly selecting 5 (400×) fields of view with an optical microscope (Nikon). The positive sections determined by two senior pathologists were used as the positive control, and the negative sections in which the primary antibody was replaced with PBS buffer were used as the negative control. The criteria for positive expression were as follows: positive expression of KIR2DL1 on the surface of CD8^+^ T cells was indicated by the colocalization of light yellow, brownish-yellow, or brown granules on the membrane of lymphocytes (CD8 and KIR2DL1) in 2 serial sections. The mean immunohistochemical score from 5 high-power fields (×400) was used as the final immunohistochemical score. Please refer to the bibliography for details [14].

### 2.7. Grouping

The group positive for Fn-induced KIR2DL1 expression on the surface of CD8^+^ T cells was composed of the tissue samples for which in three serial sections were simultaneously positive for Fn infection, CD8 expression, and KIR2DL1 expression; this group was denoted the Fn + CD8^+^KIR2DL1 positive group. Samples that did not meet these requirements of simultaneous positivity were classified as the negative group, denoted the Fn + CD8^+^KIR2DL1-negative group.

### 2.8. Statistical analysis

SPSS 26.0 software was used for statistical analysis. Measurement data are expressed as the mean ± standard deviation ($\bar{x}$ ± *s*) values, and one-way ANOVA was used to compare differences among multiple groups. Cohen's kappa coefficient was used to measure the consistency of count data, and correlation analysis was performed with the *χ*^2^ test. The Kaplan-Meier method was used to draw survival curves. The log-rank test was used to analyze differences in survival times. COX regression was used to analyze the influence of various factors on prognosis. The high expression of KIR2DL1 on the surface of CD8^+^T cells induced by Fn in ESCC tissues, gender, age, smoking, alcohol consumption, degree of differentiation, depth of infiltration, lymph node metastasis, and clinical stage were used as independent variables to conduct univariate COX regression analysis. Multivariate COX regression analysis was performed by selecting variables that were statistically associated with survival in univariate analysis and incorporating confounding factors (gender and age). *p* < .05 was considered statistically significant.

## 3. Results

### 3.1. Fn can induce KIR2DL1 expression on CD8^+^ T cells

**Figure 2.**
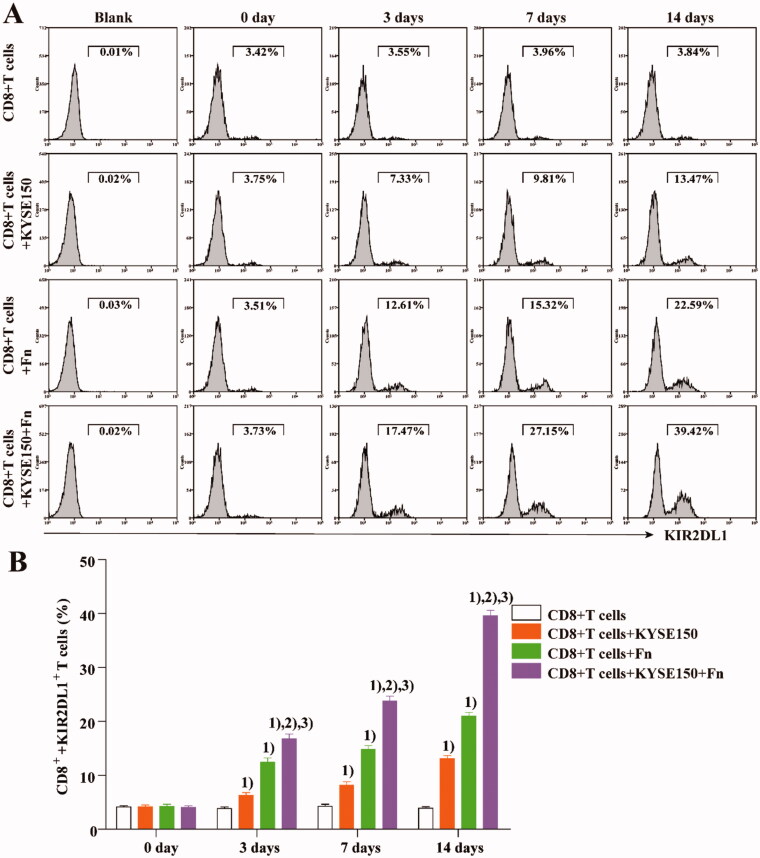
Fn can induce KIR2DL1 expression on CD8^+^ T cells. (A) The expression of KIR2DL1 on CD8^+^ T cells was detected by flow cytometry; (B) The expression of KIR2DL1 on CD8^+^ T cells among the groups at each time point was compared by one-way ANOVA; (1) Compared with the CD8^+^ T cells group, *p* < .05; (2) Compared with the CD8^+^ T cells + KYSE150 cells group, *p* < .05; (3) Compared with the CD8^+^ T cells + Fn group, *p* < .05.

CD8^+^ T cells from the peripheral blood of healthy persons were cocultured with KYSE150 cells or infected with Fn. At different time points, CD8^+^ T cells were isolated from the coculture system and incubated with an anti-KIR2DL1 antibody (Miltenyi). The expression of KIR2DL1 on CD8^+^ T cells was detected by flow cytometry (Beckman Coulter) at different time points. Figure 2 shows that compared with that in untreated CD8^+^ T cells, the expression of KIR2DL1 on the surface of CD8^+^ T cells was increased by coculture with KYSE150 cells or Fn infection. The expression of KIR2DL1 increased the most when Fn infection was combined with coculture. These results suggest that both co-culture with tumour cells or infection with Fn can increase the expression of KIR2DL1 on CD8^+^ T cells and that the combination of these treatments can increase the expression of KIR2DL1 most significantly.

### 3.2. Fn infection can reduce the sensitivity of tumour cells to CDDP

**Figure 3.**
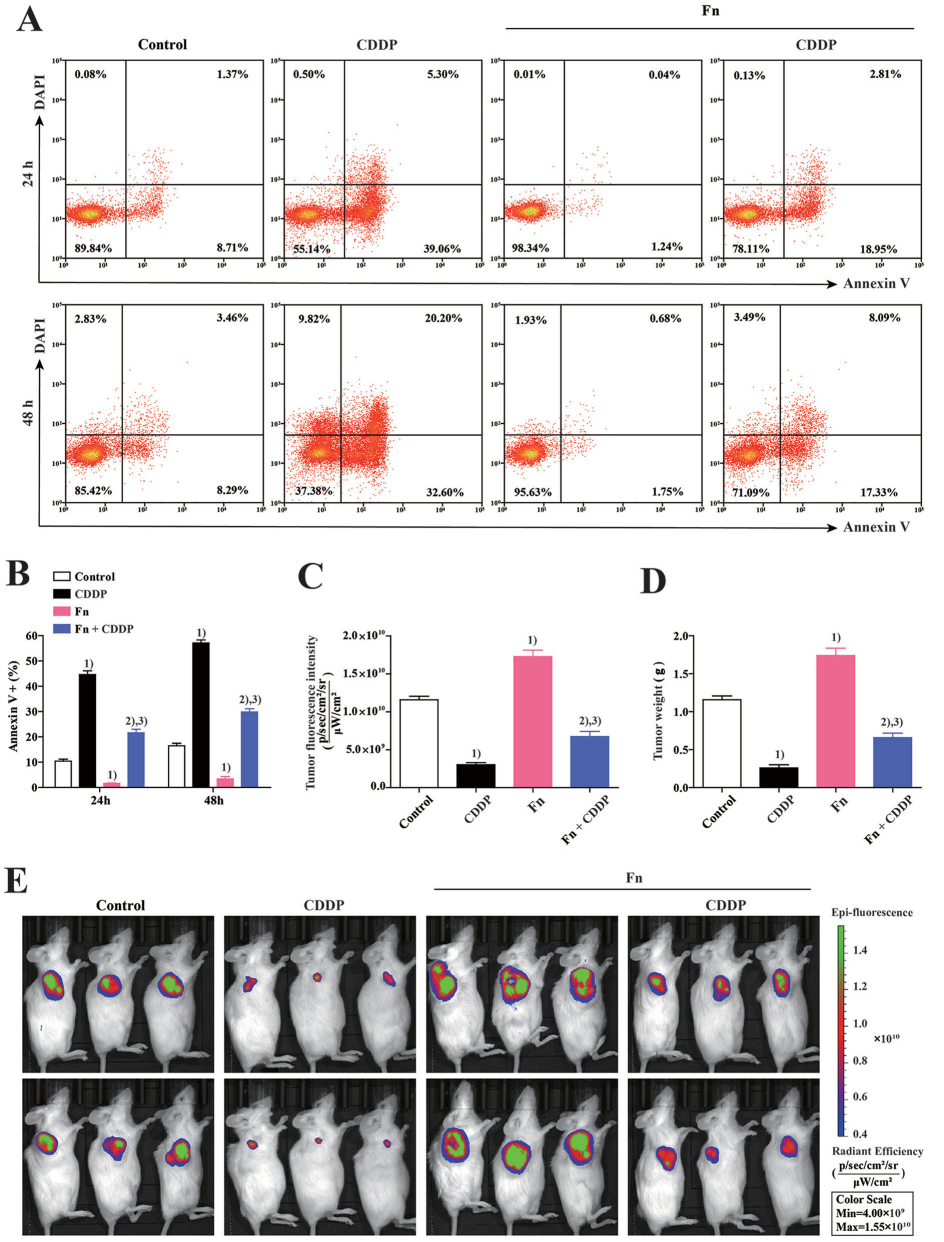
Fn infection can reduce the sensitivity of tumour cells to CDDP. (A) Apoptosis of KYSE150 cells in the co-culture system was detected by flow cytometry; (B) The apoptosis rate of KYSE150 cells was different among all groups; (C) Differences in tumour size and fluorescence intensity among groups; (D) Differences in tumour weight among groups; (1) Compared to the control group, *p* < .05; (2) Compared with the Fn group, *p* < .05; (3) Compared with the CDDP group, *p* < .05. (E) *In vivo* imaging was used to measure the tumour size in each mouse group.

To evaluate the effect of Fn infection on the CDDP sensitivity of tumour cells, a coculture system of peripheral blood CD8^+^ T cells and KYSE150 cells was established as described in Section 2.2. As shown in Figure 3(A), the coculture system was divided into four groups. As described in Section 2.3, Fn infection and CDDP (5 µg/mL, Selleck Chemicals) treatment were applied in the different groups for different periods of time (24 and 48 h). Finally, we used an apoptosis detection kit (Invitrogen) to detect the apoptosis of KYSE150 cells in each group of the coculture system by flow cytometry (Beckman Coulter) (Figure 3(B)). The results indicated that compared with that in the control group, the apoptosis rate of tumour cells in the co-culture system was significantly increased after CDDP treatment (*p* < .05) but significantly decreased after Fn infection (*p* < .05). Compared with Fn infection alone, treatment with CDDP after Fn infection significantly increased the apoptosis rate of tumour cells (*p* < .05). Compared with that in the CDDP group, the apoptosis rate of tumour cells in the Fn + CDDP group was significantly decreased (*p* < .05). These results indicate that Fn infection can reduce the sensitivity of tumour cells to CDDP. An *in vivo* model of the coculture system was established as described In Section 2.4 for verification (Figure 3(E)). (Figure 3(C,D)) results showed: Compared with that in control mice, the tumorigenic ability was obviously reduced in mice treated with CDDP (*p* < .05) but was significantly increased in mice infected with Fn (*p* < .05). Compared with that in Fn-infected mice, the tumorigenic ability in Fn-infected mice treated with CDDP was obviously reduced (*p* < .05). Compared with that in the CDDP treatment group, tumorigenicity in the Fn + CDDP treatment group was obviously reduced (*p* < .05). These results were consistent with those of the *in vitro* experiments. These results indicated that Fn infection can reduce the sensitivity of tumour cells to CDDP.

### 3.3. Detection of Fn infection and KIR2DL1 expression on CD8^+^ T cells in ESCC tissue

**Figure 4.**
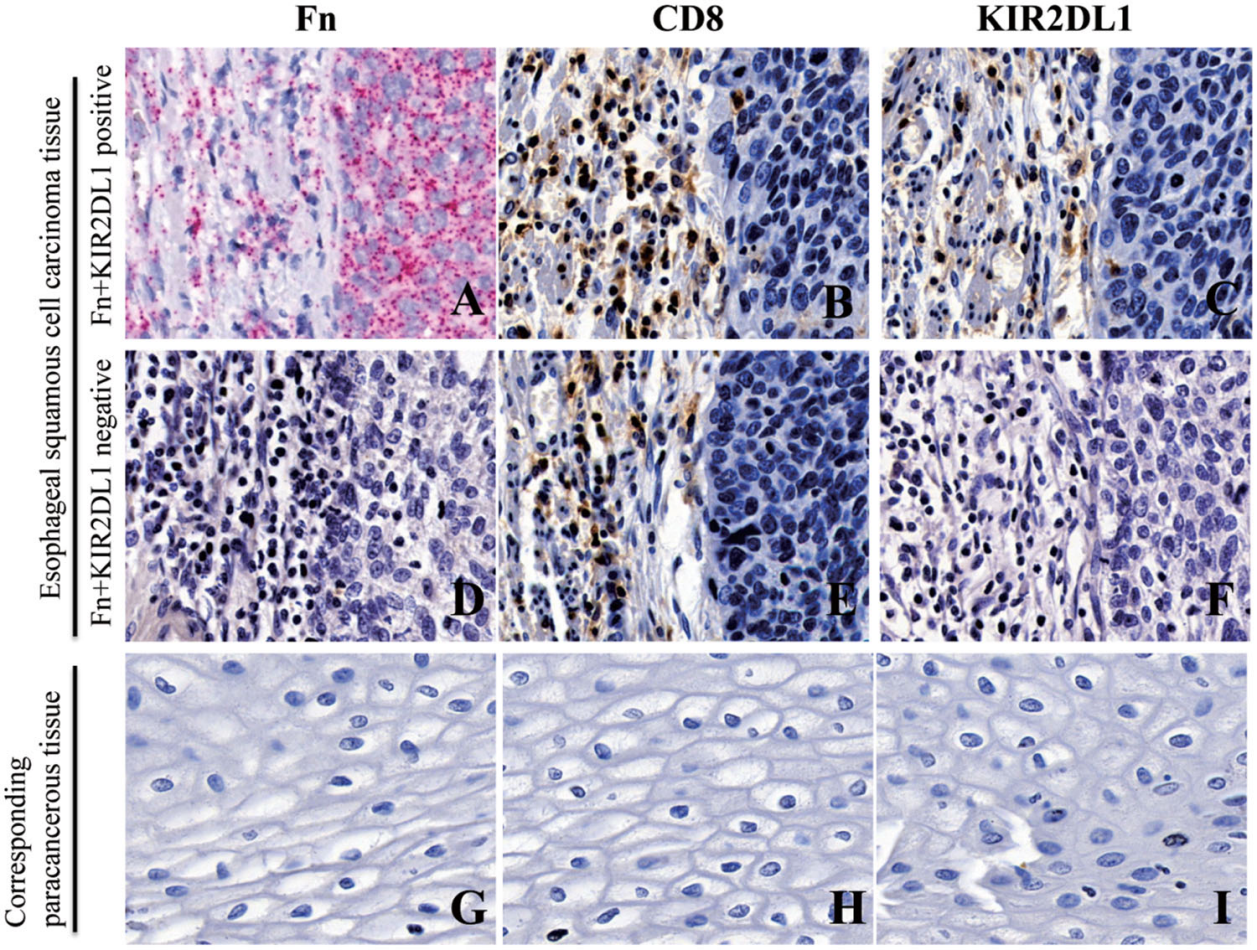
Fn infection and KIR2DL1 expression on the surface of CD8^+^ T cells in ESCC and adjacent tissues (400×). (A,D,G) Representative RNAscope images of Fn infection (16S rRNA); (B,E,H) Representative immunohistochemical images of CD8^+^ T cell infiltration; (C,F,I) Representative immunohistochemical images of KIR2DL1 expression.

Red particles were seen in the cytoplasm of cancer cells in ESCC tissues, indicating positivity for Fn infection (Figure 4(A)). In continuous sections, brownish-yellow granules appeared on the membrane of lymphocytes at the same location, indicating positive expression of KIR2DL1 on the surface of CD8^+^T cells (Figure 4(B,C)). Red particles were not seen in the cytoplasm of cancer cells in ESCC tissues, indicating negativity for Fn infection (Figure 4(D)). In continuous sections, brownish-yellow granules did not appear on the membrane of lymphocytes at the same location, indicating negative expression of KIR2DL1 on the surface of CD8+T cells (Figure 4(E,F)). The expression of KIR2DL1 on the surface of CD8^+^ T cells was consistent with Fn infection in ESCC (*p* < .05, Table 1). The positive rate of Fn infection in ESCC tissues was significantly higher than that in adjacent tissues (*p* < .05, Table 2). The positive rate of KIR2DL1 expression on CD8^+^ T cells in cancer tissues was significantly higher than that in adjacent tissues (*p* < .05, Table 3, Figure 4(G–I)).

**Table 1.** Cohen's kappa coefficients indicate the consistency between Fn infection and expression of the inhibitory receptor KIR2DL1 on the surface of CD8^+^ T lymphocytes in ESCC [*n* (%)].

	Fn		Kappa*	*p*
	Positive	Negative		
CD8^+^ T cell surface KIR2DL1				
Positive	58 (96.67)	2 (3.33)	0.929	<.001
Negative	4 (2.94)	132 (97.06)		

*Kappa coefficient >0.7, excellent; 0.4–0.7, good; <0.4, poor agreement.

**Table 2.** Comparison of the positive rates of Fn infection in cancer tissues and corresponding adjacent tissues of patients with ESCC [*n* (%)].

Group	Cancer		*χ* ^2^	*p*
	Fn (+)	Fn (−)		
Paracancerous				
Fn (+)	8 (100.00)	0 (0)	18.026	<.001
Fn (−)	54 (28.72)	134 (71.28)		

**Table 3.** Comparison of KIR2DL1 expression on the surface of CD8^+^ T cells in ESCC tissues and the corresponding paracancerous tissues [*n* (%)].

Group	Cancer		*χ* ^2^	*p*
	CD8^+^ + KIR2DL1 (+)	CD8^+^ + KIR2DL1 (−)		
Paracancerous				
CD8^+^ + KIR2DL1 (+)	6 (100.00)	0 (0)	6.88	<.001
CD8^+^ + KIR2DL1 (−)	54 (28.42)	136 (71.58)		

### 3.4. Fn-induced high expression of KIR2DL1 on CD8^+^ T cells is correlated with the clinicopathological features of ESCC patients

The results are shown in Table 4. Most ESCC patients in the Fn + CD8^+^KIR2DL1 positive group were male smokers and alcohol drinkers. The degree of differentiation was low, the degree of invasion was deep, the lymph node metastasis was early and the clinical stage was late (*p* < .05).

**Table 4.** Correlation between positivity for Fn-induced expression of the inhibitory receptor KIR2DL1 on the surface of CD8^+^ T lymphocytes and clinicopathological features of patients with ESCC [*n* (%)].

Clinicopathological feature	*n*	Fn + CD8^+^ T + KIR2DL1		$\chi^{2}$	*P*
		Positive	Negative		
Sex					
Male	128	50 (39.06)	78 (60.94)	15.883	<.001
Female	68	8 (11.76)	60 (88.24)		
Age					
≥60 years	110	30 (27.27)	80 (72.73)	0.647	.421
<60 years	86	28 (32.56)	58 (67.44)		
Smoking					
Yes	97	48 (49.48)	49 (50.52)	36.474	<.001
No	99	10 (10.10)	89 (89.90)		
Alcohol drinking					
Yes	100	50 (50.00)	50 (50.00)	40.813	<.001
NO	16	8 (50.00)	8 (50.00)		
Differentiation type					
Poorly differentiated	41	29 (70.73)	12 (29.27)	42.115	<.001
Moderately to well-differentiated	155	29 (18.71)	126 (81.29)		
Infiltration depth					
Invasion of outer membrane	139	54 (38.85)	85 (61.15)	19.658	<.001
No invasion of outer membrane	57	4 (7.02)	53 (92.98)		
Lymphatic metastasis					
Yes	96	54 (56.25)	42 (43.75)	64.179	<.001
No	100	4 (4.00)	96 (96.00)		
Clinical stage					
I/II	113	4 (3.54)	109 (96.46)	86.925	<.001
III/IV	83	54 (65.06)	29 (34.94)		

### 3.5. Cox regression analysis of prognostic factors in ESCC patients

A total of 196 ESCC patients were followed up successfully. With each factor as an independent variable, univariate COX regression analysis was carried out. The results showed that gender, smoking, alcohol consumption, degree of differentiation, depth of invasion, lymph node metastasis, clinical stage, and Fn + CD8^+^KIR2DL1 positive group were correlated with overall survival (Table 5, *p <* .05). Multivariate COX regression analysis was performed by selecting variables that were statistically associated with survival in univariate analysis (*p* < .05) and incorporating confounding factors (gender and age). The results showed that alcohol consumption, degree of differentiation, depth of infiltration, lymph node metastasis, clinical stage, and Fn + CD8^+^KIR2DL1 positive group were independent risk factors affecting the prognosis of ESCC (Table 5, *p <* .05).

**Table 5.** Cox regression analysis of prognostic factors in patients with lung adenocarcinoma.

Clinical variables	*B*	*Wald*	*HR*	95%CI		*p*
Univariate cox analysis						
Sex (male/female)	0.576	8.562	1.779	1.210	2.617	.003
Age (≥60/<60)	0.313	3.140	1.367	0.967	1.932	.076
Smoking (positive/negative)	1.084	33.222	2.956	2.045	4.273	.001
Alcohol (positive/negative)	1.219	40.277	3.384	2.322	4.931	.001
Differentiation type (poorly/moderately-well)	0.541	6.526	1.717	1.134	2.600	.011
Infiltration depth (≥Adventitia/<Adventitia)	0.625	8.288	1.867	1.221	2.857	.004
Lymph node metastasis (positive/negative)	1.028	31.744	2.796	1.955	3.999	.001
Clinical stages (III–IV/I–II)	0.972	29.734	2.644	1.864	3.750	.001
Fn + CD8^+^KIR2DL1 (positive/negative)	0.645	12.123	1.907	1.326	2.742	.001
Multivariate cox analysis						
Sex (male/female)	0.577	2.332	1.781	0.849	3.737	.127
Age (≥60/<60)	0.253	1.637	1.288	0.874	1.898	.201
Smoking (positive/negative)	0.575	1.086	1.777	0.603	5.240	.297
Alcohol (positive/negative)	2.319	11.795	10.170	2.707	38.211	.001
Differentiation type (poorly/moderately-well)	1.930	35.893	6.888	3.664	12.950	.001
Infiltration depth (≥Adventitia/<Adventitia)	0.649	8.227	1.914	1.228	2.982	.004
Lymph node metastasis (positive/negative)	0.929	22.773	2.533	1.729	3.710	.001
Clinical stages (III–IV/I–II)	0.859	18.832	2.361	1.602	3.480	.001
Fn + CD8^+^KIR2DL1 (positive/negative)	1.143	13.792	3.137	1.716	5.734	.001

### 3.5. Fn-induced high expression of KIR2DL1 on CD8^+^ T cells is correlated with the 5-year survival prognosis of ESCC patients

**Figure 5.**
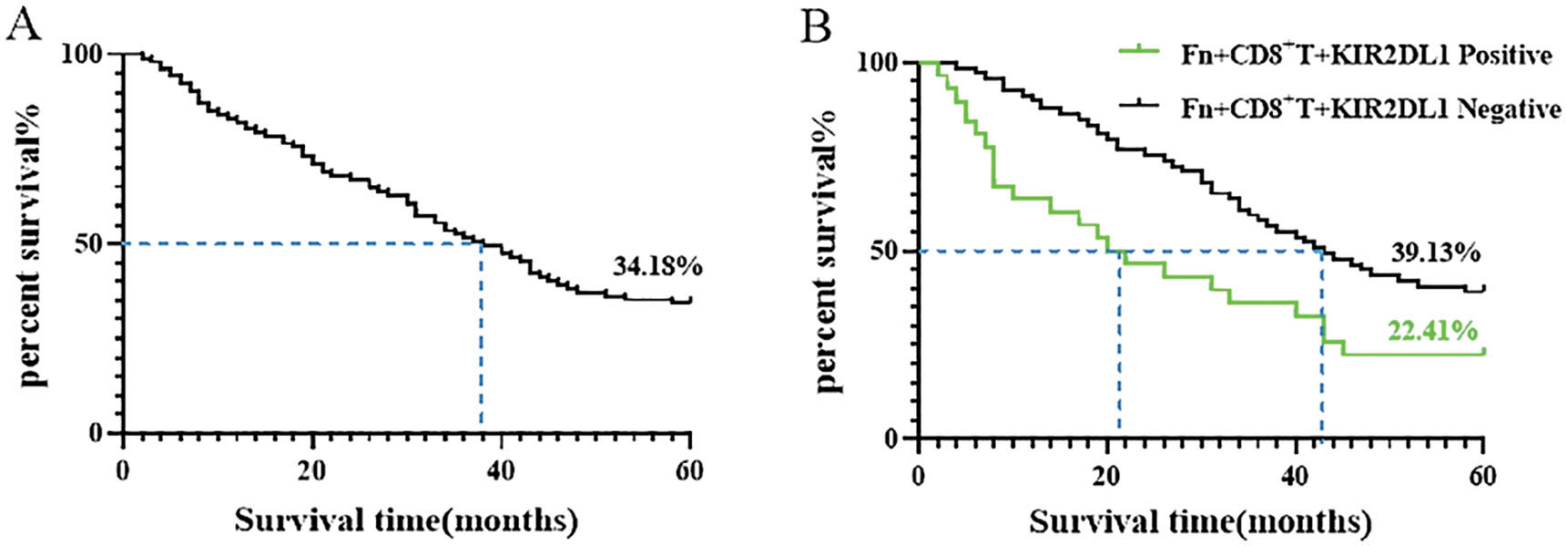
(A) Kaplan–Meier survival curve of patients with ESCC 5 years after surgical resection; (B) Kaplan–Meier 5-year survival curve of ESCC patients positive and negative for Fn-induced expression of the inhibitory receptor KIR2DL1 on the surface of CD8^+^ T lymphocytes.

The results are shown in Table 6 and Figure 5. The 5-year overall survival rate of the 196 ESCC patients was 34.18%, and the median survival time was 38.00 ± 2.69 months. In the Fn + CD8^+^KIR2DL1 positive group, the 5-year survival rate was 22.41% and the median survival time was 20.00 ± 4.28 months, significantly lower than the 39.13% and 43.00 ± 3.78 months in the Fn + CD8^+^KIR2DL1-negative group. The differences were statistically significant (*p* < .05).

**Table 6.** Mean and median survival times (months) of ESCC patients positive for Fn-induced expression of the inhibitory receptor KIR2DL1 on the surface of CD8^+^ T lymphocytes.

Fn + CD8^+^ T + KIR2DL1	Mean				Median				$\chi^{2}$	*p*
	Estimated value	Standard error	95%CI		Estimated value	Standard error	95%CI			
			Lower part	Upper part			Lower part	Upper part		
Positive	27.810	2.801	22.321	33.300	20.000	4.284	11.604	28.396	12.791	<.001
Negative	40.942	1.602	37.802	44.082	43.000	3.776	35.600	50.400		
Total	37.056	1.464	34.187	39.925	38.000	2.692	32.723	43.277		

## 4. Discussion

The prognosis of ESCC is very poor, and early diagnosis is difficult [15–18]. The 5-year survival rate of patients with advanced ESCC is extremely low [19]. It is very important to identify the aetiology of ESCC and find early diagnostic indicators and effective preventive measures. Recent studies have indicated that various pathogenic microorganisms can facilitate tumour cell immune escape by remodelling the tumour microenvironment and weakening the immune response [20,21]. *Helicobacter pylori* can induce high expression of inhibitory receptors on the surface of CD8^+^ T cells and natural killer (NK) cells and promote the malignant proliferation of gastric cancer cells [22,23]. Both hepatitis B virus and Fn can induce high expression of inhibitory receptors on the surface of CD8^+^ T cells, making them unable to perform their normal functions, leading to tumour cell escape from immune surveillance and promoting malignant progression [24,25]. Our previous study confirmed that immunosuppressive regulatory T (Treg) cells were enriched in Fn-infected ESCC tissues. Moreover, a high abundance of Treg cells can cause antitumour immune inactivation [3]. These results suggest that Fn-mediated remodelling of the tumour immune microenvironment may promote the development of ESCC.

**Figure 6.**
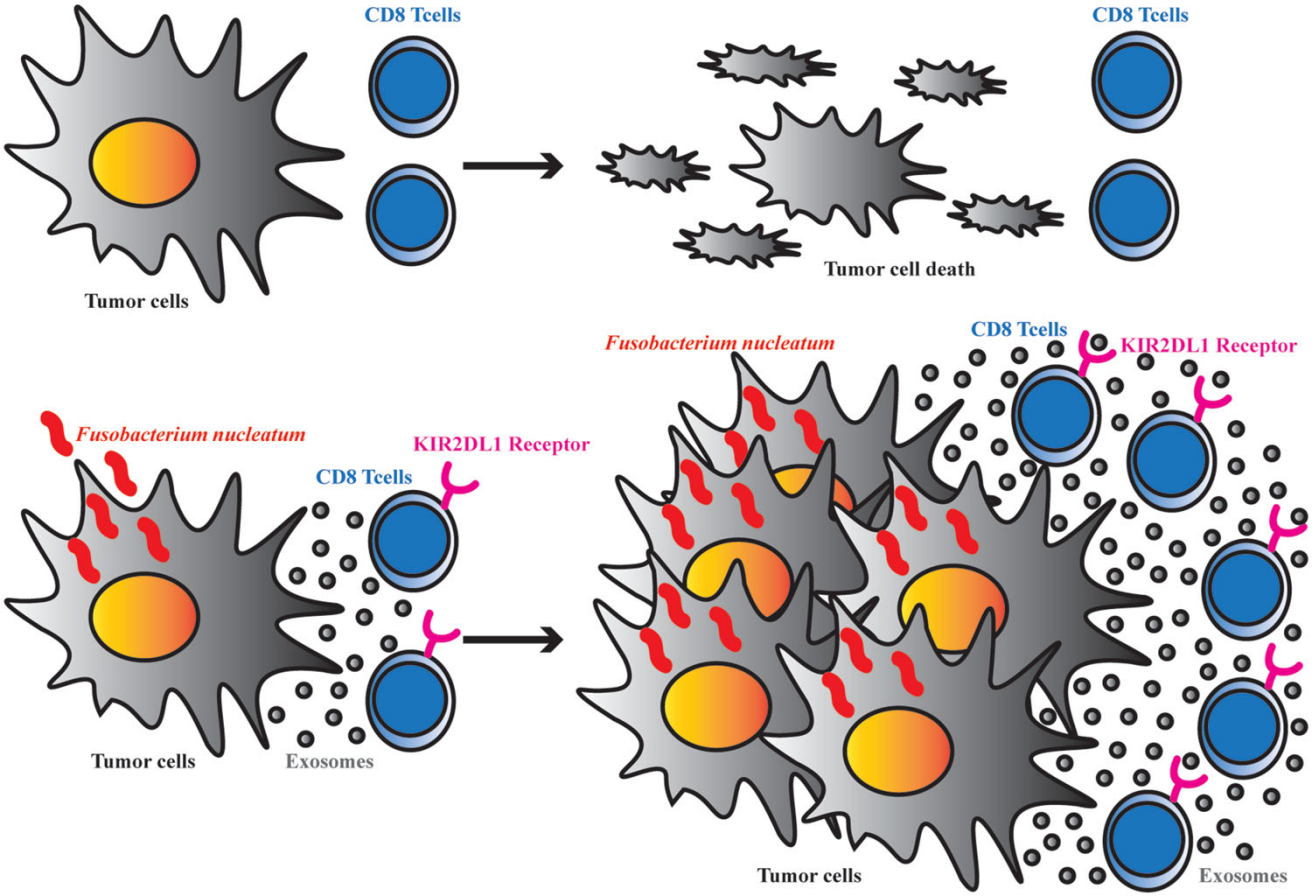
Schematic diagram.

In this study, KIR2DL1 expression on the surface of CD8^+^ T cells in the coculture system increased gradually with increasing Fn infection time, suggesting that Fn can induce high expression of KIR2DL1 on CD8^+^ T cells. In the *in vivo* and *in vitro* experiments, the apoptosis rate of tumour cells was increased and the tumorigenic ability was decreased in the CDDP group compared with the control group, suggesting that CDDP can cooperate with CD8^+^ T cells is significantly inhibiting tumour development. In the Fn group, the apoptosis rate of tumour cells was decreased and the tumorigenic ability was enhanced, suggesting that Fn weakened the ability of CD8^+^ T cells to kill tumour cells. The apoptosis rate of tumour cells was decreased and the tumorigenic ability was enhanced in the Fn + CDDP group compared with the CDDP group, suggesting that Fn infection can reduce the efficiency of the tumour cell response to CDDP by weakening the killing ability of CD8^+^ T lymphocytes. These results suggest that Fn can induce high expression of KIR2DL1 on the surface of CD8^+^ T cells and weaken their killing ability, thus facilitating tumour cell evasion of immune surveillance and decreasing the therapeutic efficacy of CDDP (see the schematic diagram of the mechanism in Figure 6). Moreover, the expression of KIR2DL1 on CD8^+^ T cells in ESCC tissues after Fn infection was significantly higher than that on CD8^+^ T cells in adjacent tissues. In addition, the expression of KIR2DL1 on the surface of CD8^+^ T cells in cancer tissues was consistent with Fn infection in cancer tissues, suggesting that the immune microenvironment of cancer tissue is more conducive to Fn survival. Fn may induce high expression of KIR2DL1 on the surface of CD8^+^ T cells to inhibit the immune response and induce tumour cell immune escape. In this study, we analyzed the correlations between Fn-induced high expression of KIR2DL1 on the surface of CD8^+^ T cells and the clinicopathological features of ESCC patients. In ESCC, Fn + CD8^+^KIR2DL1 positivity was significantly correlated with male sex, smoking, and alcohol drinking. The findings suggested that the Fn + CD8^+^KIR2DL1 positive group was predominantly composed of male patients who smoked and drank, and long-term smoking and drinking might lead to an unhealthy oral environment. This unhealthy oral environment is more prone to infection and colonization with Fn, which induces high expression of KIR2DL1 on the surface of CD8^+^ T cells. The proportion of patients in the Fn + CD8^+^KIR2DL1 positive group with poorly differentiated ESCC was higher than that of patients with moderately and well-differentiated ESCC. These results indicate that tumours with a higher degree of malignancy and their microenvironment were more conducive to Fn survival and induction of KIR2DL1 expression on the CD8^+^ T cell surface. Fn + CD8^+^KIR2DL1 positivity was obviously correlated with deep tumour invasion, positive lymph node metastasis, and more advanced clinical stage. These results indicate that Fn-induced KIR2DL1 expression on CD8^+^ T cells may promote the malignant progression of tumours. This study also found that alcohol consumption, degree of differentiation, depth of infiltration, lymph node metastasis, clinical stage, and Fn-induced CD8^+^T cells with high expression of KIR2DL1 were independent risk factors affecting the prognosis of ESCC patients, and the 5-year survival rate and median survival time of patients in the positive group were significantly lower than those in the negative group. These results suggest that Fn can induce KIR2DL1 on THE surface of CD8^+^T cells and shorten the survival of ESCC patients. Since the occurrence and development of tumours is a multifactor and multistep process, more studies are needed to prove the specific pathogenic mechanism of Fn. However, both elimination of Fn in the host and inhibition of KIR2DL1 expression on CD8^+^ T cells are important for improving the prognosis and prolonging the survival of ESCC patients.

In conclusion, Fn may induce high expression of KIR2DL1 on the surface of CD8^+^ T cells in ESCC tissues to provide a favourable microenvironment for self-sustaining infection, thus promoting tumour progression. Effective elimination of Fn and inhibition of KIR2DL1 expression on CD8^+^ T cells may prolong the survival of ESCC patients, which is very important in the clinical treatment of ESCC and has broad application prospects.

## Data Availability

The data that support the findings of this study are available on request from the corresponding author. The data are not publicly available due to privacy or ethical restrictions.
